# Hesperetin from Root Extract of *Clerodendrum petasites* S. Moore Inhibits SARS-CoV-2 Spike Protein S1 Subunit-Induced NLRP3 Inflammasome in A549 Lung Cells via Modulation of the Akt/MAPK/AP-1 Pathway

**DOI:** 10.3390/ijms231810346

**Published:** 2022-09-07

**Authors:** Punnida Arjsri, Kamonwan Srisawad, Sariya Mapoung, Warathit Semmarath, Pilaiporn Thippraphan, Sonthaya Umsumarng, Supachai Yodkeeree, Pornngarm Dejkriengkraikul

**Affiliations:** 1 Department of Biochemistry, Faculty of Medicine, Chiang Mai University, Chiang Mai 50200, Thailand; 2 Center for Research and Development of Natural Products for Health, Chiang Mai University, Chiang Mai 50200, Thailand; 3 Anticarcinogenesis and Apoptosis Research Cluster, Faculty of Medicine, Chiang Mai University, Chiang Mai 50200, Thailand; 4 Akkraratchkumari Veterinary College, Walailak University, Nakhon Si Thammarat 80160, Thailand; 5 Division of Veterinary Preclinical Sciences, Department of Veterinary Biosciences and Veterinary Public Health, Faculty of Veterinary Medicine, Chiang Mai University, Chiang Mai 50100, Thailand

**Keywords:** NLRP3 inflammasome, chronic inflammation, *Clerodendrum petasites*, hesperetin, anti-inflammation, spike glycoprotein S1, COVID-19

## Abstract

Inhibition of inflammatory responses from the spike glycoprotein of SARS-CoV-2 (Spike) by targeting NLRP3 inflammasome has recently been developed as an alternative form of supportive therapy besides the traditional anti-viral approaches. *Clerodendrum petasites* S. Moore (*C. petasites*) is a Thai traditional medicinal plant possessing antipyretic and anti-inflammatory activities. In this study, *C. petasites* ethanolic root extract (CpEE) underwent solvent-partitioned extraction to obtain the ethyl acetate fraction of *C. petasites* (CpEA). Subsequently, *C. petasites* extracts were determined for the flavonoid contents and anti-inflammatory properties against spike induction in the A549 lung cells. According to the HPLC results, CpEA significantly contained higher amounts of hesperidin and hesperetin flavonoids than CpEE (*p* < 0.05). A549 cells were then pre-treated with either *C. petasites* extracts or its active flavonoids and were primed with 100 ng/mL of spike S1 subunit (Spike S1) and determined for the anti-inflammatory properties. The results indicate that CpEA (compared with CpEE) and hesperetin (compared with hesperidin) exhibited greater anti-inflammatory properties upon Spike S1 induction through a significant reduction in IL-6, IL-1β, and IL-18 cytokine releases in A549 cells culture supernatant (*p* < 0.05). Additionally, CpEA and hesperetin significantly inhibited the Spike S1-induced inflammatory gene expressions (*NLRP3*, *IL-1β*, and *IL-18*, *p* < 0.05). Mechanistically, CpEA and hesperetin attenuated inflammasome machinery protein expressions (NLRP3, ASC, and Caspase-1), as well as inactivated the Akt/MAPK/AP-1 pathway. Overall, our findings could provide scientific-based evidence to support the use of *C. petasites* and hesperetin in the development of supportive therapies for the prevention of COVID-19-related chronic inflammation.

## 1. Introduction

Severe acute respiratory syndrome coronavirus 2 (SARS-CoV-2) infection or COVID-19 has become a serious public health problem worldwide. SARS-CoV-2 infects the human respiratory system and causes severe inflammatory responses. Two types of COVID-19-related inflammation were classified according to the chronological phase of onset. During the infection phase, hyperinflammation occurs in targeted lung tissue due to the hyper-responsiveness of the innate immune system leading to an unnecessarily high amount of pro-inflammatory cytokine production. This could lead to harmful damage to the lung tissue resulting in chronic respiratory failure or acute respiratory distress syndrome (ARDS). Another type of inflammation is the post- or after-effect of COVID-19 infection also known as post-acute COVID syndrome or long-COVID. Recent evidence has indicated that a range of symptoms could remain after the clearance of the acute infection in many people who have had COVID-19. These conditions can include fever, dyspnea, cough, skin rashes, brain fog, and chronic systemic inflammation [1]. The prevalence of long-COVID symptoms is approximately 35% in patients treated for COVID-19 on an outpatient basis but was approximately 87% of the cohorts’ hospitalized patients [2]. The dysregulated innate immune response can contribute to the clinical presentation of patients with severe COVID-19 [3]. In severe cases, patients could display ARDS with systemic inflammation, wherein lung injuries were associated with the release of inflammasome-related cytokines such as IL-6, IL-1β, and IL-18 [4]. In addition, it was reported that SARS-CoV-2 infection can activate inflammasomes, which are associated with severity in COVID-19 patients [5].

Inflammasomes are an important cellular component that can be indicative of cellular stresses and infections, which can in turn initiate inflammatory responses. Several reports have found that SARS-CoV-2 infection and the SARS-CoV-2 spike glycoprotein can promote the inflammasome activation associated with increased pro-inflammatory cytokine and chemokine release from lung and immune cells [6,7,8]. Generally, the nucleotide-binding oligomerization domain-like receptor containing pyrin domain 3 (NLRP3) inflammasome activation intensely induces cytokine production as an inflammatory response to viral infection [9]. Moreover, a previous study found that the SARS-CoV-2 spike glycoprotein promotes NLRP3 inflammasome activation and can induce hyperinflammation [10]. The NLRP3 inflammasome machinery is comprised of the adaptor molecule apoptosis-associated speck-like protein containing a caspase activation and recruitment domain (ASC) and caspase-1. After the assembly of the inflammasome complex, caspase-1 is activated by proteolytic cleavage and promotes the activation of substrates, and the inflammatory cytokine releases of IL-1β and IL-18 [11,12].

Recently, the therapeutic use of naturally occurring phytochemicals has been increasingly recognized with regard to COVID-19 outbreaks [13,14]. *Clerodendrum petasites* S. Moore (*C. petasites*) belongs to the family *Lamiaceae*. It is widely grown in India, Malaysia, and Thailand. In Thai traditional medicine, its leaves and roots are used in the treatment of fever, inflammation, skin diseases, and asthma [15,16]. In addition, *C. petasites* has generally been used in formulations of some multi-herb medicinal recipes such as the traditional formula “Ha-Rak” remedy (also known as Ben-Cha-Lo-Ka-Wi-Chian). The formulation is comprised of the five roots of *C. petasites*, *Ficus racemona* Linn, *Capparis micracantha* DC, *Harrisonia perforate* Merr, and *Tiliacora triandra* Diels plants, and was prescribed for antipyretic activity [17,18]. Many biological and pharmacological studies of *C. petasites* reported that it exhibits anti-inflammatory, antipyretic, antimicrobial and antibacterial properties [15,19]. Previous studies have found that the ethanolic extracts of *C. petasites* displayed anti-inflammatory and antipyretic properties in an in vivo model [15,20]. Additionally, *C. petasites* also contained various natural flavonoids, such as hispidulin, hesperidin, and hesperetin which are the main active compounds associated with the beneficial biological activities of the plants [21,22]. Briefly, hesperidin exhibited strong antioxidant activity and could attenuate influenza a virus (H1N1)-induced lung injury in rats through its anti-inflammatory properties [23]. Hispidulin exhibited neuroprotective effects by inhibiting neuroinflammation in LPS-induced BV2 Microglia through attenuation of Akt, NF-κB, and STAT3 activation and suppressing NLRP3-mediated pyroptosis in cerebral ischemia-reperfusion injuries [24,25]. Moreover, hesperetin was previously found to exhibit a strong anti-inflammatory effect by regulating the TLR4–MyD88–NF-κB signaling pathway in LPS-induced acute lung injuries [26]. Therefore, *C. petasites*, along with its bioactive flavonoids, can potentially be utilized as an anti-inflammatory agent against the spike glycoprotein of SARS-CoV-2 induction.

Regarding the in vitro experiment, many studies adopted the cell culture model of airway epithelial cells that express an angiotensin-converting enzyme 2 (ACE2) receptor, including human nasal epithelial cells (HNEC), human bronchial epithelial cells (HBEC), type II pneumocyte cell line (A549 cells), or the lung cancer cell line (Calu-3 cells) [27,28,29,30,31]. These cell cultures were used to investigate the mechanism of spike glycoprotein-induced inflammation and related experiments with SARS-CoV-2 induction, as SARS-CoV-2 can infect human cells through the spike glycoprotein, which is cleaved into S1 and S2 subunits, and S1 directly binds to the ACE2 receptor. It can facilitate the cell membrane and trigger lung damage by inducing inflammatory responses [32,33]. Therefore, in this study, the efficacy of the *C. petasites* extract and its active flavonoids on attenuation of the Spike S1-induced inflammasome inflammatory pathway on A549 lung cells was investigated. The outcomes of this study will provide scientific-based evidence to support the use of *C. petasites* extract and its bioactive flavonoids as therapeutic strategies for COVID-19-related chronic inflammation by targeting the NLRP3 inflammasome pathway.

## 2. Results

### 2.1. Phytochemical Characteristics and the Active Compound Identification of C. petasites Extract

In order to select a *C. petasites* extract with potential anti-inflammatory properties, we first established four *C. petasites* fractions and then determined their phytochemical characteristics and the active flavonoid compounds using the HPLC technique. Ethanolic extract of *C. petasites* (CpEE) was prepared by using 80% ethanol extraction. Accordingly, the % yield of CpEE was at 4.43% (*w/w*). The CpEE was further processed using the solvent-partitioned extraction technique to concentrate the phytochemical contents in CpEE and to obtain the *C. petasites* dichloromethane fraction (CpDM), the *C. petasites* ethyl acetate fraction (CpEA), and the *C. petasites* water fraction (Cp-H_2_O), respectively. The phytochemical contents of all *C. petasites* extracts were determined and are shown in Table 1. CpEA contained the highest total phenolic content (262.97 ± 12.70 mg of gallic acid equivalents/g extract) and the total flavonoid content (112.75 ± 4.32 mg of gallic acid equivalents/g extract) followed by CpEE, CpDM, and Cp-H_2_O. According to the literature reviews, *C. petasites* contained a wide range of phenolic and flavonoid compounds [21]. Therefore, in this study, the flavonoid compounds, hispidulin, hesperidin, and hesperetin in *C. petasites* extracts were quantified using HPLC. The concentrations of three flavonoid compounds in *C. petasites* extracts were calculated from their respective standard calibration curves and are shown in Table 1. Accordingly, hesperidin and hesperetin were identified in CpEA in high amounts at 34.98 ± 3.92 mg/g extract and 18.30 ± 0.15 mg/g extract, respectively. Whereas hispidulin was found as the major compound in CpDM at 18.82 ± 0.10 mg/g extract. When compared with CpEE, both concentrated fractions (CpEA and CpDM) contained significantly higher amounts of flavonoid compounds (*p* < 0.01). Overall, from the results, it can be concluded that the solvent partition extraction technique was found to enrich the amounts of these active compounds in *C. petasites* extracts and the extracts can be used for in vitro biological studies in further experiments.

### 2.2. Cytotoxicity of C. petasites Extracts on A549 Cells

**Figure 1:**
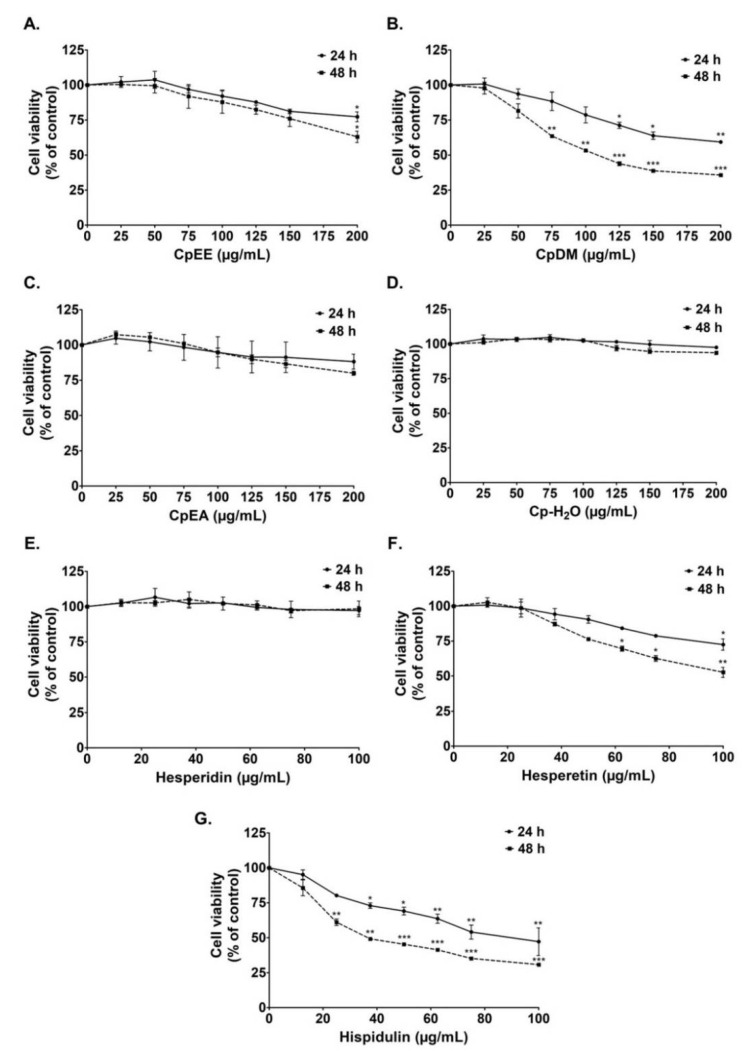
Effects of *C. petasites* extracts and its active compounds on A549 lung cell viability. Cells were treated with CpEE (**A**), CpDM (**B**), CpEA (**C**), Cp-H_2_O (**D**), hesperidin (**E**), hesperetin (**F**) and hispidulin (**G**) for 24 and 48 h. Cell survival was determined using SRB assay. Data are presented as mean ± S.D. values of three independent experiments. * *p* < 0.05, ** *p* < 0.01 and *** *p* < 0.001 vs. control.

Prior to the determination of the anti-inflammatory properties of the *C. petasites* extracts, the effects of *C. petasites* extracts and active flavonoid compounds on the A549 cells’ cytotoxicity were first determined. The effects of the *C. petasites* extracts (CpEE, CpDM, CpEA, and Cp-H_2_O) and three active compounds (hispidulin, hesperidin, and hesperetin) on A549 lung cell viability were determined by SRB assay. CpEE, CpEA, and Cp-H_2_O exhibited no cytotoxicity effects on A549 cells after 24 h and 48 h incubation, as is shown in Figure 1. Notably, CpDM exhibited a cytotoxic effect on A549 cells, as the 50% inhibitory concentration (IC_50_) was approximately 200 and 120 μg/mL for 24 and 48 h of incubation, respectively (Figure 1B). Hesperidin and hesperetin also exhibited no cytotoxicity on A549 cells after 24 h and 48 h of incubation, as is shown in Figure 1E,F. Whereas hispidulin presented a cytotoxic effect on A549 cells as the IC_50_ of hispidulin after 24 and 48 incubations was 80 and 35 μg/mL, respectively (Figure 1G).

Overall, it can be concluded that CpEE and CpEA and their active compounds, hesperidin and hesperetin, displayed no cytotoxicity effects against A549 lung cells. Therefore, the non-toxic concentrations (cell viability > 80%) of CpEE and CpEA (0–200 μg/mL), and the active compounds (0–40 μg/mL) hesperidin and hesperetin, were employed in further experiments to investigate their relevant anti-inflammatory properties on A549 cells. Notably, due to the highly cytotoxic effects of CpDM and hispidulin, they were not included in these further investigations.

### 2.3. Evaluation of CpEA- and Hesperetin-Induced Toxicity in Normal Cells

**Figure 2:**
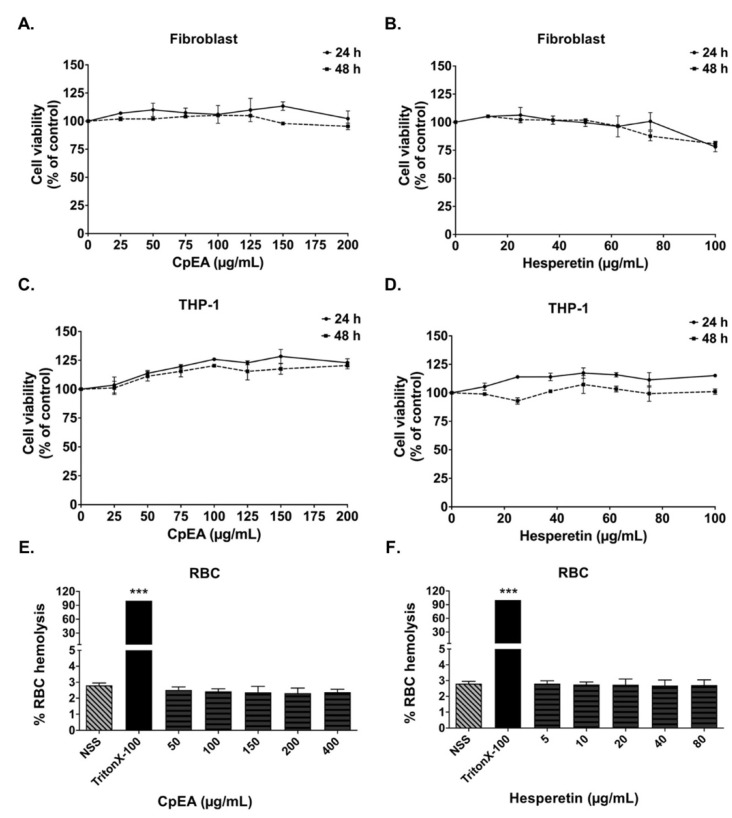
Effects of ethyl acetate fraction of *C. petasites* (CpEA) and hesperetin on normal cell viability. Cells were treated with CpEA and hesperetin for 24 and 48 h. Cell survival was determined using SRB assay for human dermal fibroblasts (**A**,**B**) and THP-1 macrophages (**C**,**D**). Effects of CpEA (**E**) and hesperetin (**F**) on red blood cells (RBCs) were determined using RBC hemolysis assay, TritonX-100 was used as positive control. Data are presented as mean ± S.D. values of three independent experiments. *** *p* < 0.001 vs. negative 0.9% normal saline (NSS) control.

Flavonoids are affordable and environmentally friendly substances with minimal or no side effects. Indeed, in our study, we determined the cytotoxicity of *C. petasites* extract and its active compound, hesperetin, on A549 cells and normal cells. As the results in Figure 1 indicate, CpEA and hesperetin had no cytotoxicity effects on A549 cells. Similar to A549 cells, CpEA and hesperetin did not induce cytotoxicity in THP-1 human macrophage cells and primary human dermal fibroblasts either at 24 or 48 h of treatment. Moreover, CpEA at concentrations of 0–200 μg/mL, and hesperetin at concentrations of 0–100 μg/mL, did not induce red blood cells (RBCs) hemolysis (Figure 2). Thus, CpEA and hesperetin demonstrated no harmful effects on normal cells.

### 2.4. Effect of C. petasites Extracts and Their Bioactive Compounds on the Inhibition of Pro-Inflammatory Cytokine Releases (IL-6, IL-1β, and IL-18) in Spike S1-Induced A549 Cells

**Figure 3:**
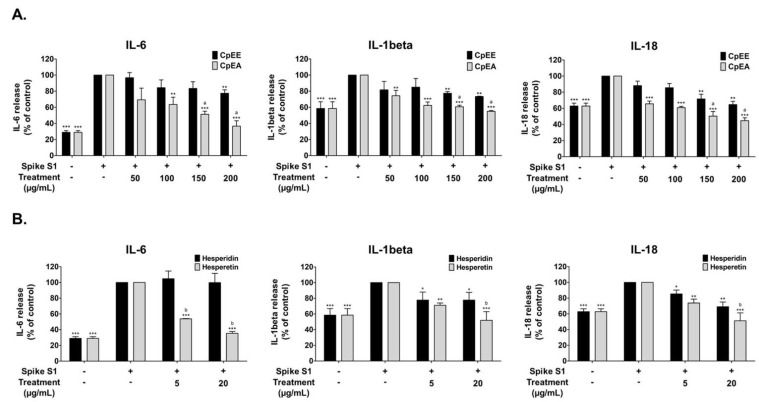
Inhibitory effects of *C. petasites* extracts and its active compounds on the pro-inflammatory cytokine release in Spike S1-induced A549 cells. A549 cells were pre-treated with *C. petasites* extracts (**A**), CpEE and CpEA, at a concentration of 0–200 μg/mL or active compounds (**B**), hesperidin or hesperetin, at a concentration of 0–20 μg/mL for 24 h. Then, the cells were exposed to Spike S1 (100 ng/mL) for 3 h. The IL-6, IL-1beta and IL-18 releases into the culture supernatant were examined by ELISA. The Spike S1-induced A549 cells are presented as 100%. Data are presented as mean ± S.D. values of three independent experiments, * *p* < 0.05, ** *p* < 0.01 and *** *p* < 0.001 vs. the Spike S1-induced control group. ^a^ *p* < 0.05 vs. CpEE at the same concentration. ^b^ *p* < 0.05 vs. hesperidin at the same concentration.

To determine the anti-inflammatory effects of *C. petasites* extracts (CpEE and CpEA) and their bioactive compounds (hesperidin and hesperetin) upon Spike S1-induction, the cytokine releases, including IL-6, IL-1β, and IL-18, into the supernatant of A549 cells were determined by ELISA. The pro-inflammatory cytokine release levels of IL-6, IL-1β, and IL-18 from Spike S1-induced A549 cells were significantly increased when compared with the non-Spike S1 group (*p* < 0.001), as is shown in Figure 3. The CpEE and CpEA treatments significantly decreased IL-6, IL-1β, and IL-18 releases from Spike S1-induced A549 cells in a dose-dependent manner (*p* < 0.001). Furthermore, CpEA exhibited stronger inhibitory effects of cytokine releases (IL-6, IL-1β, and IL-18) than CpEE (*p* < 0.05), as is shown in Figure 3A. With regard to the active compounds, hesperetin treatment significantly inhibited IL-6, IL-1β, and IL-18 releases from Spike S1-induced A549 cells in a dose-dependent manner (*p* < 0.001). Whereas hesperidin treatment significantly inhibited the IL-1β, and IL-18 releases (*p* < 0.05) from Spike S1-induced A549 cells but had no significant effect on IL-6 inhibition. As is shown in Figure 3B, when comparing the inhibitory effects of hesperetin and hesperidin on the pro-inflammatory cytokine releases, it was found that hesperetin possessed significantly more potent cytokine inhibition effects (IL-6, IL-1β, and IL-18) than hesperidin at concentrations of 5 or 20 μg/mL (*p* < 0.05). Overall, it can be concluded that CpEA (compared with CpEE) and hesperetin (compared with hesperidin) exhibited greater anti-inflammatory properties upon Spike S1 induction through a significant reduction in IL-6, IL-1β, and IL-18 cytokine releases in A549 cell culture supernatant.

### 2.5. Effect of C. petasites Extracts and Hesperetin on Inhibition of Pro-Inflammatory Cytokines (IL-1β, and IL-18) and NLRP3 Gene Expressions in Spike S1-Induced A549 Cells

**Figure 4:**
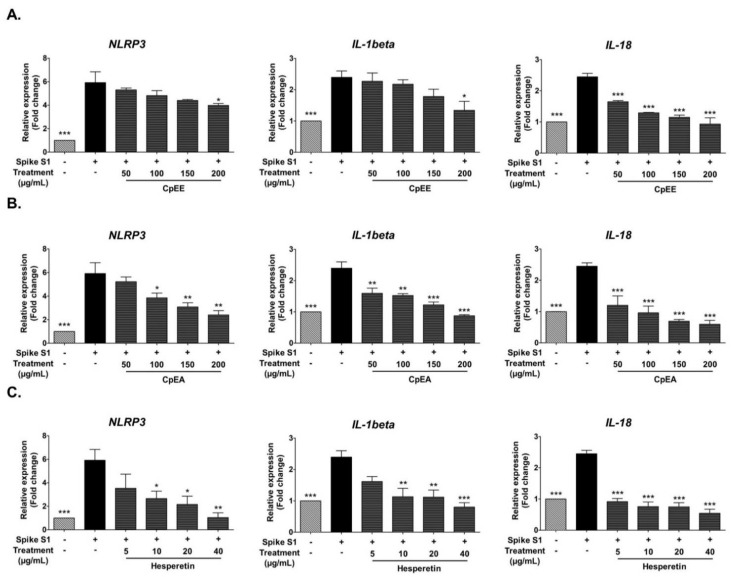
Inhibitory effects of *C. petasites* extracts and hesperetin on the *NLRP3*, *IL-1β* and *IL-18* gene expression in Spike S1-induced A549 cells. A549 cells were pre-treated with CpEE (**A**), CpEA (**B**) at a concentration of 0–200 μg/mL and hesperetin (**C**) at a concentration of 0–40 μg/mL for 24 h. Then, the cells were exposed to Spike S1 (100 ng/mL) for 3 h. The mRNA expressions were determined using RT-qPCR. Data are presented as mean ± S.D. values of three independent experiments, * *p* < 0.05, ** *p* < 0.01 and *** *p* < 0.001 vs. the Spike S1-induced A549 cells.

In addition to pro-inflammatory cytokine release, previous studies have reported that the NLRP3 inflammasome pathway activated by Spike S1 led to an increase in pro-inflammatory cytokine gene expressions, including *NLRP3, IL-1β* and *IL-18* [34,35,36,37]. Thus, the effects of the *C. petasites* extracts, CpEE and CpEA, as well as hesperetin on *NLRP3*, *IL-1β*, and *IL-18* mRNA expressions in Spike S1-induced A549 cells, were also determined using RT-qPCR. The *NLRP3*, *IL-1β*, and *IL-18* mRNA levels significantly increased in Spike S1-induced A549 cells when compared with the non-Spike S1 group (*p* < 0.001), as is shown in Figure 4. The *NLRP3, IL-1β*, and *IL-18* mRNA levels in Spike S1-induced A549 cells were significantly decreased in a dose-dependent manner upon CpEE and CpEA treatments (Figure 4A). As expected, CpEA exhibited stronger inhibitory effects on mRNA levels of inflammatory cytokines and *NLRP3* than CpEE (*p* < 0.05). Hesperetin, the flavonoid compound that is enriched in CpEA, demonstrated pronounced inhibitory effects on IL-1β, and IL-18, and NLRP3 gene expressions in Spike S1-induced A549 cells in a dose-dependent manner (*p* < 0.05). From the results, it can be concluded that CpEA and hesperetin significantly inhibited the Spike S1-induced inflammatory gene expressions (*NLRP3*, *IL-1β*, and *IL-18*). Therefore, CpEA and hesperetin were the anti-inflammatory candidates to be further investigated in the inhibition of the NLRP3 inflammasome pathways against Spike S1-induced A549 cells.

### 2.6. Inhibitory Effects of CpEA and Hesperetin on the NLRP3 Inflammasome Pathway in Spike S1-Induced A549 Cells

**Figure 5:**
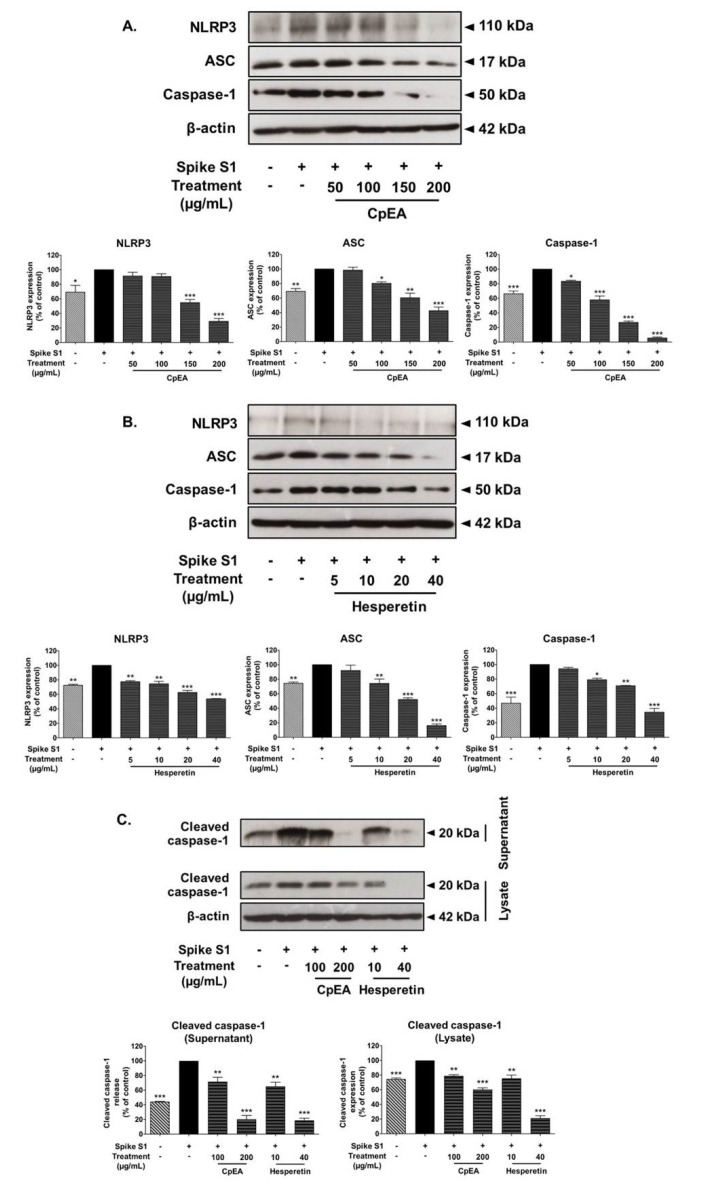
CpEA and hesperetin inhibited the NLRP3 inflammasome pathway in Spike S1-induced A549 lung cells. A549 lung cells were pre-treated with CpEA at a concentration of 0–200 μg/mL or hesperetin at a concentration of 0–40 μg/mL for 24 h, and then exposed to Spike S1 (100 ng/mL) for 3 h. The inhibitory effects of CpEA (**A**) and hesperetin (**B**) on the expression of NLRP3, ASC, and caspase-1 proteins in A549 cells. The inhibitory effects of CpEA and hesperetin on the expressions of cleaved caspase-1 in culture supernatant and cell lysate from A549 cells (**C**). The data is displayed in Western blot and band density measurements. The Spike S1-induced A549 cells are presented as 100% of control. Data are presented as mean ± S.D. values of three independent experiments, * *p* < 0.05, ** *p* < 0.01 and *** *p* < 0.001 vs. the Spike S1-induced A549 cells.

The NLRP3 inflammasome complex is comprised of NLRP3, ASC, and pro-caspase-1. Activation of the inflammasome pathway leads to protein–protein interaction between NLRP3 and ASC. This can cause the ASC to associate with pro-caspase-1, which leads to activation of caspase-1 followed by pro-inflammatory cytokine secretion (IL-1β and IL-18) [11]. Therefore, inhibition of the NLRP3 inflammasome pathway could potentially be the targeted pathway for Spike S1-induced inflammation. Accordingly, the inhibitory effects of CpEA and hesperetin on the NLRP3 inflammasome machinery protein expressions upon Spike S1-induced A549 cells were determined by Western blot analysis. The results demonstrated that the NLRP3, ASC, and caspase-1 protein expressions in Spike S1-induced A549 cells were significantly increased when compared with the non-Spike S1 group, as is shown in Figure 5 (*p* < 0.01). The CpEA and hesperetin treatments significantly decreased NLRP3, ASC, and caspase-1 protein expressions in Spike S1-induced A549 cells in a dose-dependent manner (*p* < 0.001, band density measurements), as shown in Figure 5A,B, respectively. Additionally, CpEA and hesperetin treatments significantly decreased the expression levels of the cleavage form of caspase-1 (cleaved caspase-1) in both cell lysate and cell culture supernatant samples as determined by the Western blot technique (Figure 5C).

Overall, from the results, it can be indicated that CpEA and its active compound, hesperetin, were partially responsible for the anti-inflammation properties of Spike S1-induced A549 cells via inhibition of the expressions of NLRP3 inflammasome machinery proteins and the cleavage form of caspase-1, which would then lead to a decrease in pro-inflammatory cytokine releases (IL-1β and IL-18) in both gene and protein levels.

### 2.7. Inhibitory Effects of CpEA and Hesperetin on the Akt/Erk/c-Jun Signaling Pathway in Spike S1-Induced A549 Cells

**Figure 6:**
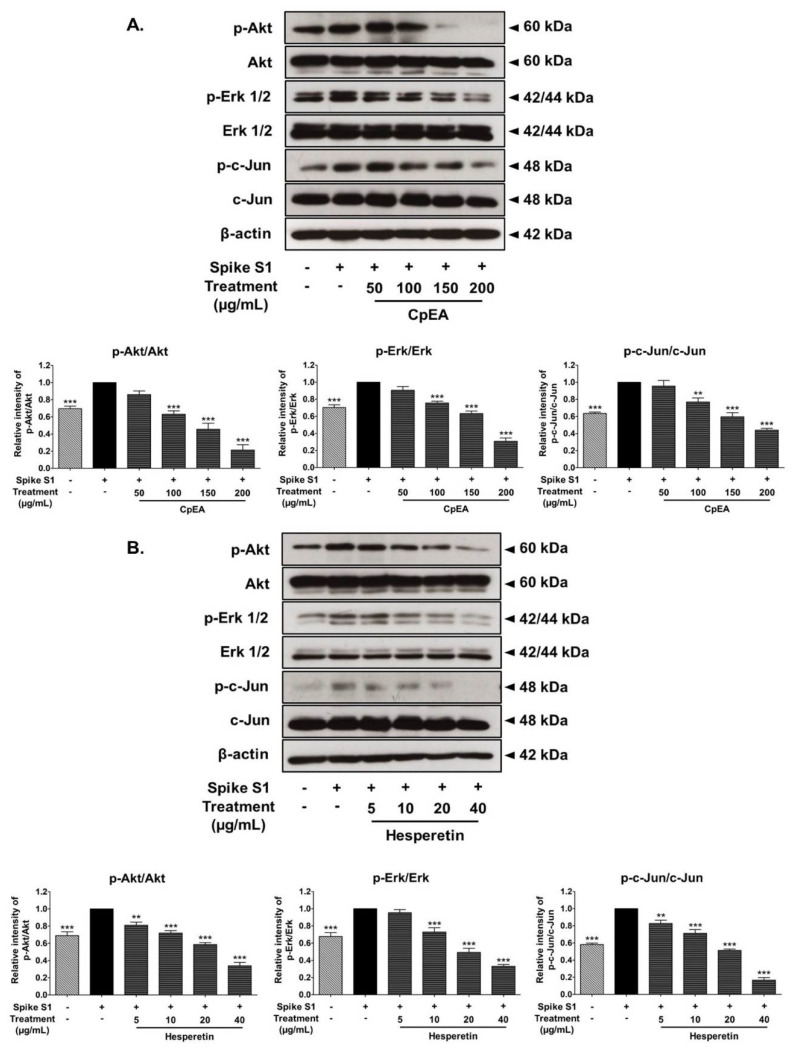
CpEA and hesperetin inactivated the Akt/Erk/c-Jun signaling pathway in Spike-S1-induced A549 cells. A549 lung cells were pre-treated with CpEA at a concentration of 0–200 μg/mL or hesperetin at a concentration of 0–40 μg/mL for 24 h, and then exposed to Spike S1 for 3 h. The inhibitory effects of CpEA (**A**) and hesperetin (**B**) on the phosphorylation of Akt, Erk1/2, and c-Jun proteins in A549 cells were displayed in Western blot and band density measurements. The Spike S1-induced A549 cells are presented as 100% of control. Data are presented as mean ± S.D. values of three independent experiments, ** *p* < 0.01 and *** *p* < 0.001 vs. the Spike S1-induced A549 cells.

In order to investigate how CpEA and hesperetin inhibited the NLRP3 inflammasome upon Spike S1 induction in A549 cells, we determined the protein expressions in the MAPK signaling pathway using the Western blot technique. Previous studies reported that during SARS-CoV-2 infection, Spike S1 could activate the Akt and MAPK signaling pathway, as well as activate the transcription factor, AP-1, leading to the NLRP3 inflammation activation and the release of pro-inflammatory cytokines [38,39]. Therefore, the effects of CpEA and hesperetin on the activation of the Akt/MAPK/c-Jun axis upon Spike S1-induced A549 cells were investigated. Accordingly, the phosphorylation of the Akt, Erk1/2 and c-Jun proteins was significantly increased in Spike S1-induced A549 cells, as is shown in Figure 6 (*p* < 0.05, band density measurement). The results also found that CpEA and hesperetin treatment significantly inhibited the phosphorylation of the Akt, Erk1/2, and c-Jun proteins when compared with the Spike S1-induced group, as shown in Figure 6A,B (*p* < 0.05, band density measurement). Overall, from the results, it can be inferred that CpEA and hesperetin treatments could attenuate the Spike S1-induced NLRP3 inflammasome inflammation through the inhibition of the Akt/MAPK/AP-1 axis and resulting in a decrease in inflammatory cytokine releases of IL-1β and IL-18. 

## 3. Discussion

**Figure 7:**
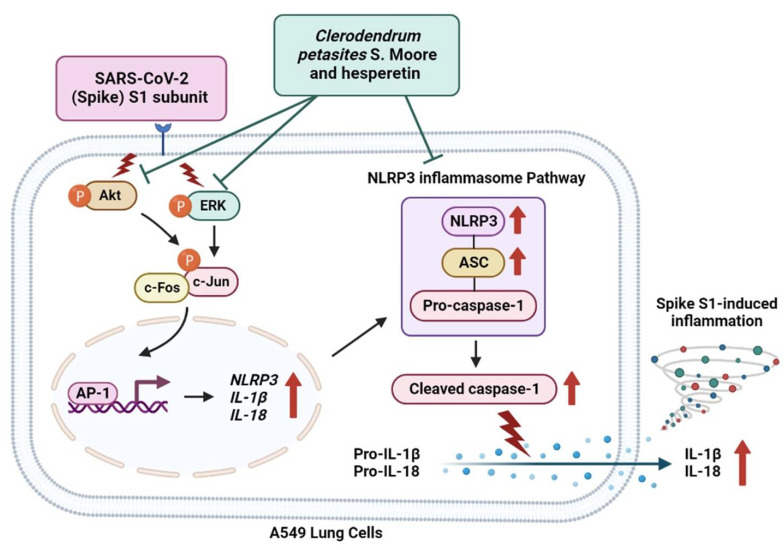
Schematic concluding mechanism of hesperetin-enriched fraction from *Clerodendrum petasites* S. Moore (CpEA) and hesperetin-attenuated Spike S1-induced NLRP3 inflammasome inflammation through the inactivating of Akt/Erk/c-Jun signaling pathway in A549 cells.

The concluding mechanism of CpEA and hesperetin inhibiting Spike-S1-induced inflammation in A549 cells is shown the Figure 7. The inflammatory conditions caused by COVID-19 manifest as a broad spectrum of symptoms ranging from sub-chronic inflammation with no symptoms to severe pneumonia that may evolve into life-threatening acute respiratory distress syndrome [5]. Specifically, the spike glycoprotein component of SARS-CoV-2 (SP) appears to share antigenic epitopes with human molecular chaperons resulting in the activation of toll-like receptors (TLRs) and downstream inflammatory pathways leading to an uncontrollable release of inflammatory cytokines. The inflammatory induction by SP is not only affected during the cytokine storm in patients during the critical period of COVID-19, but as previous findings have indicated, patients can also develop a chronic condition characterized by fatigue and neuropsychiatric symptoms, termed long-COVID. The spike protein of SARS-CoV-2 can remain in the blood circulation for 2–3 weeks after the resolved infection and can be allocated to certain organs. SP can act as a foreign antigen that triggers the lung epithelial cells and immune cells to initiate an inflammatory response. This could eventually lead to the chronic or sub-chronic systemic inflammation that was observed in COVID-19 patients during the post-infection phase [3,12]. With regard to our in vitro investigation of the SP-induced inflammatory mechanism, as well as the therapeutic potential of the *C. petasites* extract and its active compounds, we selected A549 cells as our cell culture model because ACE2-expressed airway epithelial cells were used in the experiments of previous studies that focused on SARS-CoV-2 infection and inflammation [35,36,37]. Accordingly, these aforementioned studies used the same A549 cells to represent the lower respiratory system or the alveolar epithelial cells that were induced by the spike glycoprotein. Consequently, the A549 cells could then activate inflammatory signaling resulting in the release of pro-inflammatory cytokines.

Nowadays, many studies have confirmed the role of the spike glycoprotein of SARS-CoV-2 on the inflammatory induction during COVID-19 infections. Briefly, the spike glycoprotein S1 was found to trigger an inflammatory response in human PBMC, monocyte, and lung epithelial cell lines [34,35,36,40]. Additionally, in our recent study, 100 ng/mL of the spike glycoprotein S1 was able to induce NLRP3 cytokine releases, including IL-6, IL-1β, and IL-18, in both gene and protein levels for A549 lung epithelial cells and PMA-treated THP-1 macrophages [37]. Therefore, in this study, we employed a dosage of the SARS-CoV-2 spike glycoprotein S1 subunit (S1) that was used in previously published data to investigate the anti-inflammatory properties of two potential flavonoid candidates (hesperetin and hesperidin) obtained from the root extracts of *Clerodendrum petasites* S. Moore (*C. petasites*).

*C. petasites* has been prescribed in Thai traditional medical practices for over 30 years in the treatment of various ailments including asthma, inflammation, fever, cough, vomiting, and skin disorders. The phytochemicals derived from this plant were identified as phenolic acids, flavones (hispidulin), flavone glycosides (hesperidin), and aglycone (hesperetin) [21]. However, the pharmacological activities reported in both in vitro and in vivo experiments have mostly been reported from the crude extracts of the plant. The root extract of *C. petasites* was able to inhibit the production of pro-inflammatory markers, nitric oxide, and prostaglandin E2 in lipopolysaccharide (LPS)-stimulated murine RAW 264.7 macrophages [41]. The methanol extract of *C. petasites* possessed inhibitory activity in the acute phase of inflammation in a dose-related manner, as was observed in ethyl phenylpropionate-induced ear edema and carrageenin-induced hind paw edema in rats [15]. On the other hand, many commercially available pure compounds found in *C. petasites*, including hispidulin, hesperetin, and hesperidin, were acknowledged for their potent medicinal effects, such as anti-cancer and anti-inflammatory activities [21,22,35]. Briefly, hispidulin exhibited potent anticancer activity by activating ER stress in human NCI-H460 and A549 cells as well as in an in vivo models [42]. Hesperetin suppressed inflammatory responses in LPS-induced RAW 264.7 cells via the inhibition of NF-κB and the activation of the Nrf2/HO-1 pathways [43]. Furthermore, hesperidin inhibited inflammatory responses in the human osteoarthritis (OA) model by inhibiting the activation of the NF-κB signaling pathway [44].

Taken together, *C. petasites* extracts can be utilized as complementary medicine and/or in the pharmacological development of new drugs derived from the phytochemicals present within these extracts. In our study, in order to achieve the most benefit from *C. petasites* extract, we employed the solvent-partitioned extraction technique to obtain a concentrated ethyl acetate fraction of *C. petasites* (CpEA) that contained significantly higher amounts of hesperidin and hesperetin flavonoids than the ethanolic extract of *C. petasites*. Our findings coincided with those of previous studies which reported that hesperetin, hesperidin, and hispidulin were the major bioactive compounds present in the *C. petasites* extract. There are several active compounds obtained from herb extracts that are known to possess anti-inflammatory properties [13]. Flavonoids are affordable and environmentally friendly substances with minimal or no side effects. Indeed, in our study, we determined the cytotoxicity of *C. petasites* extract and its active compound, hesperetin, on A549 cells and normal cells. The results found that CpEA and hesperetin demonstrated no harmful effects on normal cells.

Since the COVID-19 pandemic has affected people throughout the world, it is necessary to investigate all possible treatment options for COVID-19. Flavonoids, which are among the most important plant substances found in nature, could be employed to prevent chronic inflammatory conditions in long-COVID [45]. In our study, hesperidin and hesperetin were determined to be the predominant phytochemicals found in the *C. petasites* ethyl acetate fraction. With regard to COVID-19, several review articles have predicted the anti-SARS-CoV-2 infection activity of the flavonoid family, including hesperidin, as it could inhibit the viral replication of SARS-CoV-2 at the early stage of virus infection [46]. Moreover, according to the in silico studies employing hesperetin and hesperidin as the potential anti-viral agents against COVID-19, hesperetin was found to bind the two cellular proteins: transmembrane serine protease 2 (TMPRSS2) and angiotensin-converting enzyme 2 (ACE2), which are required for the cellular entry of SARS-CoV-2 [47]. Additionally, hesperetin and hesperidin suppressed the infection of VeroE6 cells using lentiviral-based pseudo-particles with wild types and variants of spike proteins, by blocking the interaction between the spike protein and cellular receptor ACE2 and reducing ACE2, and TMPRSS2 expression [48]. These findings supported that hesperetin and hesperidin could be considered potential agents to prevent SARS-CoV-2 infection. Nevertheless, there are a limited number of studies that have examined the effects of flavonoids on the inhibition of SP-induced inflammation as well as its responsible anti-inflammatory pathway. Herein, our study focused on the inhibitory effects of plant flavonoids on Spike S1-induced inflammation via the NLRP3 inflammasome pathway and related molecules in the regulation of cytokine storm conditions, which could be relevant to the prevention of inflammation-related long-COVID symptoms.

Hesperetin is an aglycone metabolite of hesperidin with high bioavailability. While hesperidin is a common flavanone glycoside. These compounds have previously been found in citrus fruits and *C. petasites*. With regard to the anti-inflammatory properties of the ethyl acetate fraction of *C. petasites* (CpEA) and its active flavonoids, our study demonstrated that CpEA and hesperetin exhibited significant anti-inflammatory properties upon Spike S1 exposure. Noticeably, the anti-inflammatory properties of hesperetin upon Spike S1 induction were significantly higher than those of hesperidin, as evidenced by the more potent inhibition of inflammatory cytokine releases (IL-6, IL-1β, and IL-18) for both the gene and protein levels in A549 cells. This finding was consistent with other studies which reported that hesperetin was recognized for its stronger antioxidant and anti-inflammation properties than its sugar moiety form, hesperidin [49,50,51]. However, the inflammation model employed in the previous studies mostly involved inflammatory inducers to trigger the canonical inflammatory pathways but not the NLRP3 inflammasome pathway.

As a basis of the pulmonary inflammatory response, it was postulated that lung epithelial cells could express the NLRP3 inflammasome, which was identified as one of the most detrimental signaling molecules in lung inflammatory conditions [52,53]. Moreover, NLRP3 is known to coordinate the uncontrolled inflammatory pathway referred to as the NLRP3 inflammasome. Previous studies have found that the spike glycoprotein from SARS-CoV-2 can activate TLRs and result in the downstream activation of multiple inflammatory signaling pathways (MAPK, NLRP3, and JAK-STAT) and activation of transcription factors, such as NF-κB, and AP-1, leading to the inflammatory genes’ transcription of *NLRP3, IL-6, pro-IL-1β*, and *pro-IL-18* [38,39]. The inflammasome complex is comprised of NLRP3, ASC, and zymogen pro-caspase-1 machinery proteins. Predominantly, the NLRP3 inflammasome responds to intracellular damage that is induced by pathogens. With regard to the mechanism of the NLRP3 inflammasome, once the protein complex has formed, the inflammasomes activate caspase-1 (cleaved caspase-1), which proteolytically activates the pro-inflammatory cytokines leading to the functional output of inflammasome activation which includes the cytokine releases of IL-1β and IL-18 [54,55,56,57]. Therefore, the findings of our study indicate that NLRP3 inflammasome activation upon Spike S1 induction, as a consequence of the formation of three inflammasome components (NLRP3, ASC, and pro-caspase 1), was inhibited by CpEA and hesperetin. We measured the protein expression of the inflammasome component in cell lysate and measured the cytokine release of IL-1β, and IL-18 in the cell culture supernatant in order to confirm the anti-inflammatory properties of CpEA and hesperetin. Moreover, the cleaved caspase-1 levels in the cell lysate and the culture supernatant were also found to have decreased with CpEA and hesperetin.

In our study, it was determined that Spike S1 exposure in A549 lung cells resulted in an increase in inflammatory gene expressions (*NLRP3*, *IL-1β*, and *IL-18*) and inflammatory cytokine releases (IL-6, IL-1β, and IL-18). Furthermore, CpEA and its active flavonoid, hesperetin, strongly inhibited an inflammatory response upon Spike S1-induction in both gene levels and cytokine releases that are involved in the NLRP3 inflammasome pathway. In addition to A549 cells, we also found that CpEA and hesperetin exhibited anti-inflammatory properties upon Spike S1-induction in normal THP-1 macrophages. The culture supernatant of Spike S1-induced THP-1 cells was analyzed for cytokine releases (IL-6, IL-1β, and IL-18) by ELISA testing. Consequently, it was found that CpEA and hesperetin significantly inhibited IL-6, IL-1β, and IL-18 cytokine releases (Figure S1).

Previously, it was found that the expression of NLRP3 and IL-1β, and the cleavage of caspase-1, was significantly elevated following LPS treatment accompanied by greater activation of mitogen-activated protein kinase (MAPK) signaling in both in vitro and in vivo studies [8]. Additionally, the blocking of p38 MAPK signaling through the inhibitor SB203580 significantly suppressed acute lung injuries and excessive lung inflammation in vivo, which was consistent with the reduced expression of the NLRP3 inflammasome and IL-1β [58]. Therefore, we hypothesized that CpEA and hesperetin could inhibit the Spike S1-induced inflammation through the inhibition of the MAPK pathway, as well as by inhibiting the expression of the inflammasome machinery proteins (NLRP3, ASC, and caspase-1) that could result in the attenuation of the inflammatory response mediated by the spike protein. According to our results, CpEA and hesperetin significantly inhibited the phosphorylation of the Akt, Erk1/2, and c-Jun proteins in Spike S1-induced inflammation. Moreover, CpEA and hesperetin could inhibit the expressions of inflammasome machinery proteins, including NLRP3, ASC, and caspase-1 (both pro- and cleavage forms). Therefore, it can be inferred that CpEA and hesperetin could attenuate the inflammatory responses upon Spike S1-induction through the inhibition of the Akt/MAPK/AP-1 axis together with the inhibition of the NLRP3 inflammasome pathway in A549 cells.

Overall, this study highlighted the anti-inflammatory properties of a flavonoid-containing plant and its active flavonoids against SARS-CoV-2 spike glycoprotein-induced inflammation in A549 lung cells. Remarkably, the anti-inflammatory effects of hesperetin could offer an advantage to the aglycone forms of flavonoids. Notably, aglycone flavonoids are high in bioavailability, which could maintain their biological activity when administrated to the body [48,59]. Nevertheless, further studies on the anti-inflammatory properties of *C. petasites* and hesperetin in normal lung tissues or the acute toxicity testing in animal models should be examined in order to identify any possible harmful effects. Our findings displayed significant evidence to support the role of IL-1β and NLRP3-dependent inflammasome activation in the pathogenesis of spike glycoprotein induced-inflammation in COVID-19 infections. The information obtained from this study could be a valuable source of evidence to support the targeting of the NLRP3 inflammasome pathway in developing preventive strategies for COVID-19-related inflammation. 

## 4. Materials and Methods

### 4.1. Chemical and Reagents 

Standard Hesperidin, hesperetin, hispidulin were purchased from MedChemExpress (Monmouth Junction, NJ, USA). Recombinant human coronavirus SARS-CoV-2 Spike Glycoprotein S1 (Active) (ab273068) was purchased from Abcam (Cambridge, UK). Dulbecco’s Modified Eagle Medium (DMEM) was purchased from Gibco (Grand Island, NY, USA). Fetal bovine serum (FBS) was purchased from Thermo Scientific (Waltham, MA, USA). Sulforhodamine B reagent, and anti-β-actin were obtained from Sigma-Aldrich (St. Louis, MO, USA). TRI reagent^®^ was purchased from Merck Millipore (Billerica, MA, USA). ReverTra Ace^®^ qPCR Master Mix was purchased from Toyobo Co., Ltd. (Osaka, Japan). SensiFAST SYBR Lo-ROX Kit was purchased from Meridian Bioscience^®^ (Cincinnati, OH, USA). The anti-caspase-1 (p50 and p20), anti-NLRP3, anti-ASC, anti-p-Akt, anti-p-Erk1/2, anti-p-c-Jun, anti-Akt, anti-Erk1/2, anti-c-Jun primary antibodies and horseradish peroxidase-conjugated anti-mouse- or anti-rabbit-IgG were purchased from Cell Signaling Technology (Danvers, MA, USA).

### 4.2. Herb Materials

*C. petasites* S. Moore. was harvested in 2021 from a local farm located in Phitsanulok Province, Thailand. The voucher specimen of *C. petasites* (No. RT048) was certified by the herbarium at the Flora of Thailand, Faculty of Pharmacy, Chiang Mai University, Thailand.

### 4.3. Preparation of C. petasites Root Extracts and Solvent-Partitioned Extraction Technique

Initially, 800 g of the dried *C. petasites* roots were soaked in 12 L of 80% (*v*/*v*) ethanol and were mixed using digital overhead stirrer (IKA^®^ RW 20, Staufen, Germany) at 256× *g* rpm for 48 h at room temperature. After filtration, the extract was evaporated using a rotary vacuum evaporator at 56 °C (Buchi^®^, Flawil, Switzerland) to obtain ethanolic extract. The ethanolic extract was resuspended in 540 mL of deionized water (DI H_2_O) and then freeze-dried to obtain the *C. petasites* ethanolic extract (CpEE). For the solvent-partitioned purification step as modified from previously described protocol [60], the ethanolic extract of *C. petasites* (23 g) was dissolved in 1000 mL of DI H_2_O and subsequently partitioned with 1000 mL of hexane. The hexane was then separated and evaporated. 

Afterward, the water fraction was partitioned with a dichloromethane:water fraction at a ratio of 1:1 (2 times). Next, the dichloromethane fraction was collected, evaporated, and lyophilized, and named the *C. petasites* dichloromethane fraction (CpDM). After that, the water fraction was then partitioned again with an ethyl acetate:water fraction at a ratio of 1:1 (2 times). The ethyl acetate fraction was then collected, evaporated, and lyophilized to finally obtain the *C. petasites* ethyl acetate fraction (CpEA). Finally, the residual fraction was collected, evaporated, and lyophilized, and named the *C. petasites* water fraction (Cp-H_2_O). CpEE, CpDM, CpEA and Cp-H_2_O were kept at −20 °C for further experimentation.

### 4.4. Total Phenolic Content

The total phenolic content of *C. petasites* extracts was determined by the modified Folin–Ciocalteu assay as previously described [60]. A total of 400 µL of each extract were mixed with 300 µL of Folin–Ciocalteu reagent and incubated at room temperature for 3 min. Next, 300 µL of sodium carbonate (Na_2_CO_3_) were added to the samples and incubated for next 30 min. After incubation, the samples were measured by spectrophotometry at 765 nm. The total phenolic content was shown as milligrams of gallic acid (GA) equivalents per gram of extract (mg GAE/g extract).

### 4.5. Total Flavonoid Content

Total flavonoid contents were measured using the aluminum chloride (AlCl_3_) colorimetric assay with slight modifications [60]. Each concentration of the *C. petasites* extracts (250 µL) was mixed with 5% NaNO_2_ (125 µL) for 5 min. Next, 10% AlCl_3_ (125 µL) was added to the mixture. After 5 min of incubation, 1.0 mL NaOH was added, and the mixture was incubated for 15 min at room temperature. The absorbance of the mixture was measured at 510 nm using a spectrophotometer and then compared with the standard catechin. The total flavonoid content was expressed as mg catechin equivalents (CE) per gram of extract (mg CE/g extract).

### 4.6. Identification of Active Compounds Using HPLC

Flavonoid active compounds in the *C. petasites* extracts were determined using HPLC (Infinity 1260, Agilent Technologies, Santa Clara, CA, USA) with reversed-phase C18 column (Zorbax Eclipse Plus C18, 250 mm × 4.6 mm, 5µm) and pre-column (Zorbax Eclipse Plus-C18, 12.5 mm × 4.6 mm, 5 µm). The HPLC condition was modified using previously described protocol [61]. The mobile phase was composed of mobile phase A (0.1% acetic acid in distilled water) and mobile phase B (acetonitrile) under gradient conditions. Pre-injection time was set as 80%A: 20%B. The following gradient elution was set as follows: 0–10 min, from 80 to 70% of A; 10.01–20 min, from 70 to 10% of A; 20.1–40 min, stable at 10% of A, and 40.1–45 min, from 10 to 80% of A. The detection wavelength was 280 nm. The flow rate was set to 1.0 mL/min for 45 min. The peak area was calculated and compared with the standard to establish the concentration for each detected compound (mg/g extract).

### 4.7. Cell Cultures

The human lung adenocarcinoma cell line (A549) (CCL-185™) was obtained from American Type Culture Collection (ATCC) (Manassas, VA, USA). The A549 cells were cultured in DMEM supplemented with 10% FBS, 2 mM L-glutamine, 50 U/mL penicillin, and 50 μg/mL streptomycin. Cells were maintained in a 5% CO_2_ humidified incubator at 37 °C. 

THP-1 cells (TIB-202^TM^) were purchased from the ATCC and using an RPMI 1640 medium supplemented with 10% FBS, 2 mM L-glutamine, 50 U/mL penicillin, and 50 μg/mL streptomycin at 37 °C in a humidified 5% CO_2_ atmosphere. THP-1 macrophage differentiation was induced via a 24 h exposure to 10 ng/mL of PMA in DMSO followed by the previously described protocol [62]. Briefly, Cells used for the resting condition were kept in the presence of PMA for 24 h in normal growth medium. The media was then changed to PMA-free RPMI during a resting stage for another 24 h. PMA-treated macrophages were maintained in a 5% CO_2_ humidified incubator at 37 °C.

Primary human skin fibroblasts were aseptically isolated from an abdominal scar after a surgical procedure involving a cesarean delivery at the surgical operation room of Chiang Mai Maharaj Hospital, Chiang Mai University, Chiang Mai, Thailand (Study code: BIO-2558-03549 approved by Medical Research Ethics Committee, Chiang Mai University). The fibroblast cells were isolated as previously described [63]. The fibroblasts were cultured in DMEM supplemented with 10% FBS, 2 mM L-glutamine, 50 U/mL penicillin, and 50 μg/mL streptomycin. Cells were maintained in a 5% CO_2_ humidified incubator at 37 °C.

### 4.8. Cell Cytotoxicity Assay

Cytotoxicity of the *C. petasites* extract and the active compounds against A549 and THP-1 cells were measured using SRB assay as was previously described [37,64]. Briefly, A549 cells (4 × 10^3^ cells/well) and THP-1 cells (6.5 × 10^3^ cells/well) were seeded in a 96-well plate and incubated at 37 °C in 5% CO_2_ overnight. After that, the cells were treated with various concentrations (0–200 μg/mL) of each *C. petasites* extract or its active compounds (0–100 μg/mL) for 24 and 48 h. After incubation, 10% (*w*/*v*) of trichloroacetic acid (100 mL) was added to the cells and incubated at 4 °C for 1 h. The medium was removed, and the cells were rinsed with slow-running tap water. Then, 0.054% (*w*/*v*) SRB solution (100 mL) was added to each well and the cells were incubated for 30 min at room temperature. The SRB solution was removed, and the cells were then washed 4 times with 1% (*v*/*v*) acetic acid. Cells were then allowed to dry at room temperature. The dye was dissolved with a 10 mM Tris-based solution (pH 10.5). The absorbance for SRB assay was measured at 510 nm using a microplate reader. Cell viability was calculated compared to control and interpreted as the % of control.

### 4.9. Red Blood Cells (RBCs) Hemolysis 

To determine the effects of *C. petasites* extract and hesperetin on the human red blood cells (RBCs). The RBCs hemolysis induction assay was performed accordingly previously described protocol [63,65]. Human blood samples were obtained from blood bank laboratory Maharaj Hospital, Chiang Mai, Thailand which remained in laboratory and cannot be identified as hosts. Briefly, packed RBCs were diluted in 0.9% Normal saline solution (NSS) to 5% RBC suspension. The 300 μL of 5% RBCs suspension were incubated with or without various concentrations (0–200 μg/mL) of *C. petasites* extract and hesperetin (0–20 μg/mL) in a 37 °C water bath for 3 h. NSS and 0.5% triton-X 100 were used as negative and positive controls, respectively. After 3 hours, the supernatant was collected by centrifugation at 4400× *g* rpm for 5 minutes at room temperature and hemoglobin concentrations were measured by spectrophotometry at 540 nm. The concentration of cell-free hemoglobin in each sample was assessed according to the hemoglobin standard curve.

### 4.10. Determination of Cytokine Release

The pro-inflammatory cytokine releases, IL-6, IL-1β and IL-18, in the cultured supernatants were determined using an ELISA kit (Biolegend, San Diego, CA, USA), followed using manufacturer’s instructions. A549 cells (3 × 10^5^ cells/well) were seeded in a 6-well plate. After overnight incubation, the cells were pre-treated with various concentrations of *C. petasites* extracts (0–200 μg/mL) or active compounds (0–20 μg/mL) for 24 h and then were induced by Spike S1 (100 ng/mL) for further 3 h. The cultured supernatant was collected for ELISA testing. The cytokine release into the culture supernatant was calculated and compared for each standard curve. 

### 4.11. Expression of IL-1β, IL-18 and NLRP3 Genes by RT-qPCR Analysis

In order to determine *IL-1β*, *IL-18* and *NLRP3* gene expressions, A549 cells were pre-treated with various concentrations of *C. petasites* extracts (0–200 μg/mL) or hesperetin (0–40 μg/mL) for 24 h and then induced with Spike S1 (100 ng/mL). After 3 h of incubation, total mRNA was isolated using TRI reagent^®^. The concentration and purity of total RNA were detected using NanoDrop™ 2000/2000c Spectrophotometers (Thermo Fisher Scientific, Waltham, MA, USA) (A260/A280 > 1.8 indicating pure RNA). The cDNA was obtained via reverse transcription using a Mastercycler^®^ nexus gradient machine (Eppendorf, GA, Germany). Quantitative real-time PCR technique was determined using a qRT-PCR ABITM 7500 Fast and 7500 real-time PCR machine (Thermo Fisher Scientific, Waltham, MA, USA). Gene expressions were analyzed using QuantStudio 6 Flex real-time PCR system software (Applied Biosystems, Waltham, MA, USA). The 2^−ΔΔCT^ method with normalization to *GAPDH* and controls was used for calculation of results. All primer sequences used in this study were as follows: *IL-1β* forward, 5′-ATGATGGCTTATTACAGTGGCAA-3′, reverse, 5′-GTCGGAGATTCGTAGCTGGA-3′; *IL-18* forward, 5′-AAACTATTTGTCGCAGGAATAAAGAT-3′ reverse, 5′-GCTTGCCAAAGTAATCTGATTCC-3′; *NLRP3* forward, 5′-AAGGGCCATGGACTATTTCC-3′ reverse, 5′-GACTCCACCCGATGACAGTT-3′ and *GAPDH* forward, 5′-TCAACAGCGACACCCAC-3′ reverse, 5′-GGGTCTCTCTCTTCCTCTTGTG-3′ (Humanizing Genomics Macrogen, Geumcheon-gu, Seoul, Korea) [66,67,68].

### 4.12. Western Blot Analysis

In order to determine the effects of CpEA or hesperetin on NLRP3 inflammasome pathway and molecular mechanism in Spike S1-induced A549 cells, Western blot analysis was performed. The A549 cells were pre-treated with various concentrations of CpEA (0–200 μg/mL) or hesperetin (0–40 μg/mL) for 24 h. After that, the cells were induced with Spike S1 (100 ng/mL) for 3 h. The cells were collected and lysed using RIPA buffer. The protein concentration was determined using the Bradford method. The whole-cell lysate was subjected to 8–12% SDS-PAGE. Separated proteins were transferred into nitrocellulose membranes. Membranes were blocked with 5% bovine serum albumin (BSA) in 0.5% TBS-tween. After that, the membranes were washed with 0.5% TBS-Tween. Then, membranes were further incubated overnight with the primary antibody at 4 °C. Next, the membranes were washed 4 times with 0.5% TBS-Tween followed by incubating with horseradish peroxidase-conjugated anti-mouse or rabbit-IgG depending on the primary antibody at room temperature for 2 h and were then washed 4 times with 0.5% the TBS-Tween. Bound antibodies were detected using the chemiluminescent detection system and then exposed to X-ray film (GE Healthcare Ltd., Little Chalfont, UK). Equal values of protein loading were confirmed as each membrane was stripped and re-probed with anti-β-actin antibody. Band density levels were analyzed using IMAGE J 1.410.

### 4.13. Statistical Analysis

All experiments were carried out in triplicate independent experiments to confirm reproducibility, and the data were presented as mean ± standard deviation (mean ± S.D.) values. Statistical analysis was analyzed with Prism version 8.0 software using independent t-test and one-way ANOVA with Dunnett’s test. Statistical significance was determined at *p < 0.05, **p < 0.01 and ***p < 0.001 ^a^ *p* < 0.05 vs. CpEE at the same concentration. ^b^ *p* < 0.05 vs. hesperidin at the same concentration.

## 5. Conclusions

COVID-19-related inflammation has now become a highly concerning health issue in the post-COVID-19 era. The activation of the NLRP3 inflammasome by the spike glycoprotein of SARS-CoV-2 can be considered a potential target in the development of COVID-19 supportive therapies. The *C. petasites* ethyl acetate fraction, containing a high amount of hesperetin, displayed significant anti-inflammatory properties. Furthermore, *C. petasites* ethyl acetate fraction and hesperetin could inhibit SARS-CoV-2 spike glycoprotein S1 subunit-induced inflammation in A549 lung cells via inhibition of the Akt/MAPK/AP-1 axis and the NLRP3 inflammasome pathway, leading to the suppression of inflammatory cytokines releases. The results obtained from this study can be applied in COVID-19 supportive therapy via the inhibition of the NLRP3 inflammasomes pathway by *C. petasites* and hesperetin that could result in the prevention of inflammatory responses upon the long-term inflammatory induction initiated by the spike glycoprotein of SARS-CoV-2.

## Figures and Tables

**Table 1:** Phytochemical characteristics and the identification of active compounds in *C. petasites* extracts.

*C. petasites* Extracts	Polyphenols (mg/g Extract)		Active Compounds (mg/g Extract)		
	Total Phenolic Content	Total Flavonoid Content	Hesperidin	Hispidulin	Hesperetin
CpEE	80.72 ± 3.03	34.12 ± 0.37	4.99 ± 0.88	2.79 ± 0.18	1.03 ± 0.05
CpDM	180.22 ± 13.87	83.48 ± 3.61	8.91 ± 0.28	18.82 ± 0.10 ***	7.79 ± 0.29
CpEA	262.97 ± 12.70 ***	112.75 ± 4.32 ***	34.98 ± 3.92 ***	2.80 ± 0.16	18.30 ± 0.15 ***
Cp-H_2_O	70.63 ± 3.00	24.57 ± 0.13	21.19 ± 5.44	ND	ND

ND = Not detectable, *** *p* < 0.001 vs. the others *C. petasites* extracts. Data are presented as mean ± S.D. values of three independent experiments.

## Data Availability

Not applicable.

## Supplementary Materials

The following supporting information can be downloaded at: https://www.mdpi.com/article/10.3390/ijms231810346/s1.
