# Supplementary material for: Hesperetin from Root Extract of Clerodendrum petasites S. Moore Inhibits SARS-CoV-2 Spike Protein S1 Subunit-Induced NLRP3 Inflammasome in A549 Lung Cells via Modulation of the Akt/MAPK/AP-1 Pathway
Source: Int J Mol Sci. 2022 Sep 7;23(18):10346. doi: 10.3390/ijms231810346 (PMC9498987; doi:10.3390/ijms231810346)
Supplement: Supplementary file 1 [file ijms-23-10346-s001.zip › ijms-1831719-supplementary.pdf]

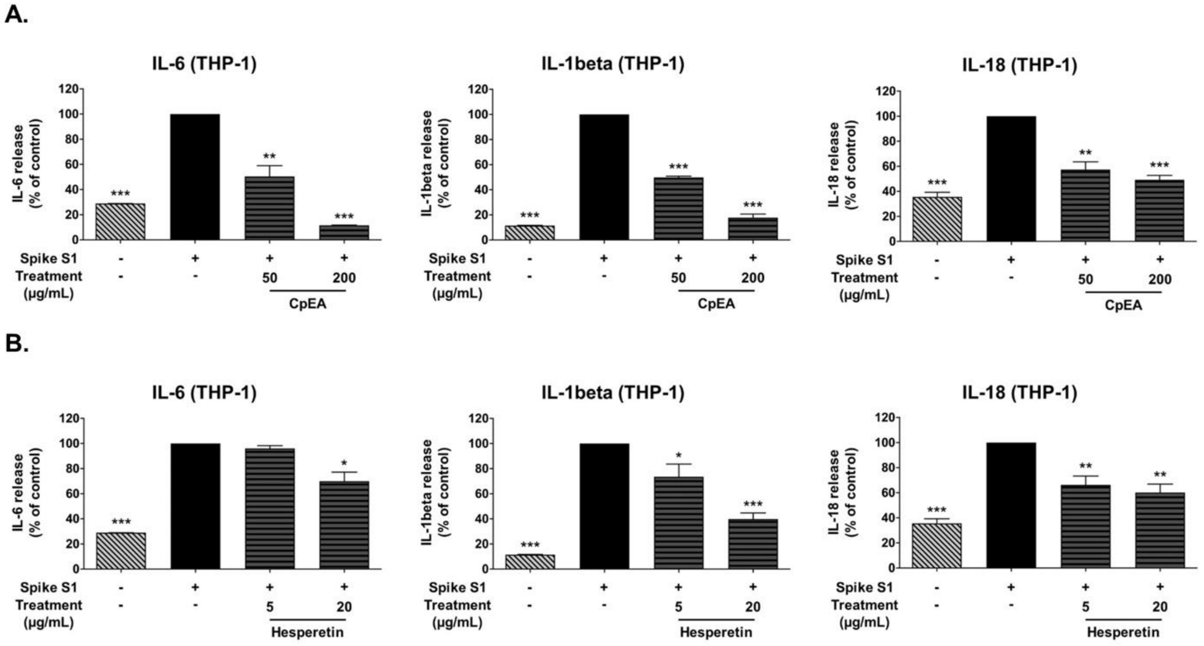

Figure S1. Inhibitory effects of ethyl acetate fraction of *C. petasites* (CpEA) and hesperetin on Spike S1 induced inflammation in THP-1 macrophages. Cells were treated with CpEA (50 and 200 ug/mL) and hesperetin (5 and 20 ug/mL) for 24 hours. Then, the cells were exposed to Spike S1 (100 ng/mL) for 3 hours. The IL-6, IL-1beta and IL-18 releases into the culture supernatant were examined by ELISA. The Spike S1-induced THP-1 cells are presented as 100%. Data are presented as mean ± S.D. values of three independent experiments, * $p < 0.05$, ** $p < 0.01$ and *** $p < 0.001$ vs. the Spike S1-induced control group.
